# Complex management of extensive perivisceral aortic thrombus and associated chronic kidney disease in a 70-year-old woman

**DOI:** 10.1016/j.jvscit.2026.102461

**Published:** 2026-08-22

**Authors:** Madison Kropp, Max Murray-Ramcharan, Amarseen Mikael, S. Christopher Frontario, Thomas Bernik

**Affiliations:** Department of Vascular Surgery, Englewood Health, Englewood, NJ

**Keywords:** Aortic thrombus, Perivisceral, Thoracic aortobirenal, Bypass, Aortic endarterectomy, Aortic dissection

## Abstract

Severe perivisceral aortic thrombus is a rare cause of embolic organ dysfunction with limited treatment guidance. We report a 70-year-old woman with progressive renal failure due to extensive perivisceral and renal artery thrombus. Given high embolic risk, endovascular therapy was deferred, and open aortic endarterectomy was performed. Persistent renal artery compromise necessitated intraoperative conversion to extra-anatomic thoracobirenal bypass. Postoperatively, renal function normalized with durable graft patency at 1 year. A concomitant descending thoracic aortic dissection was managed conservatively. This case highlights the limitations of endovascular therapy in extensive aortic thrombus and supports individualized open revascularization strategies in select patients.

Arterial embolic disease is a significant cause of ischemia, with common sources including intracardiac thrombi, debris from aortic aneurysms, aortic arch atherosclerotic plaques, and paradoxical emboli from deep vein thrombosis. Perivisceral aortic thrombus is a less common source of embolization but carries significant consequences, including distal arterial occlusion, organ failure, and limb loss. These thrombi typically form over areas of aortic wall pathology, such as aneurysms or complex plaques.^1^ However, primary aortic mural thrombi have also been reported in the absence of underlying aortic or cardiac disease.^2^^,^^3^

The increasing use of computed tomography angiography has led to more frequent detection of these lesions.^4^ Despite this, there are no well-established guidelines for the management of perivisceral sources of embolic disease.^3^ Current treatment strategies include anticoagulation, antiplatelet therapy, thrombolysis, thrombectomy, and both endovascular and open surgical approaches.^5^

This case highlights the challenges in managing extensive perivisceral aortic thrombus with renal involvement, as well as the role of open surgical revascularization and management of associated complications. The patient has provided written consent for use of case details and images within this article.

## Case report

A 70-year-old woman, former smoker, with a history of hypertension, hyperlipidemia, chronic kidney disease, a 4-cm abdominal aortic aneurysm, and known bilateral renal artery stenosis with aortic mural thrombus on anticoagulation presented with worsening renal function. Over a 6-month period, she progressed from stage IIIa to stage IV chronic kidney disease. Computed tomography angiography demonstrated an extensive perivisceral and perirenal aortic thrombus with near-complete occlusion of the left renal artery (Fig 1), corroborating the clinical picture of progressive ischemic nephropathy. Preoperative angiography was performed to further delineate the extent of left renal artery occlusion, provide supplementary views for possible endovascular intervention, and continuously evaluate whether intervention was necessary (Fig 2). Given the extensive mural thrombus and visceral involvement, surgical repair was recommended to limit further solid organ dysfunction. Because of hostile perivisceral aortic segments with perceived high risk of embolic phenomena, endovascular techniques were not pursued. An open thoracoabdominal approach was selected to allow adequate proximal control, given concern for a compromised supraceliac aorta. The patient was positioned in a right semilateral decubitus position, and a thoracoabdominal incision at the seventh intercostal space was performed to expose the descending thoracic and abdominal aorta via a retroperitoneal approach. The aorta was exposed from the descending thoracic segment to the infrarenal level, and the celiac artery, superior mesenteric artery, and bilateral renal arteries were isolated. After proximal and distal control, a longitudinal aortotomy was performed in the perivisceral aorta, revealing extensive chronic thrombus with acute components extending into the visceral and renal arteries. Endarterectomy of the aorta, superior mesenteric artery, and bilateral renal arteries was performed; however, disease-free end points at the renal artery orifices were not able to be achieved. Given this proximal stenosis and the extent of the atherosclerotic disease, isolated endarterectomy was insufficient to revascularize the kidneys. An intraoperative decision was therefore made to proceed with extra-anatomic revascularization.

**Fig 1:**
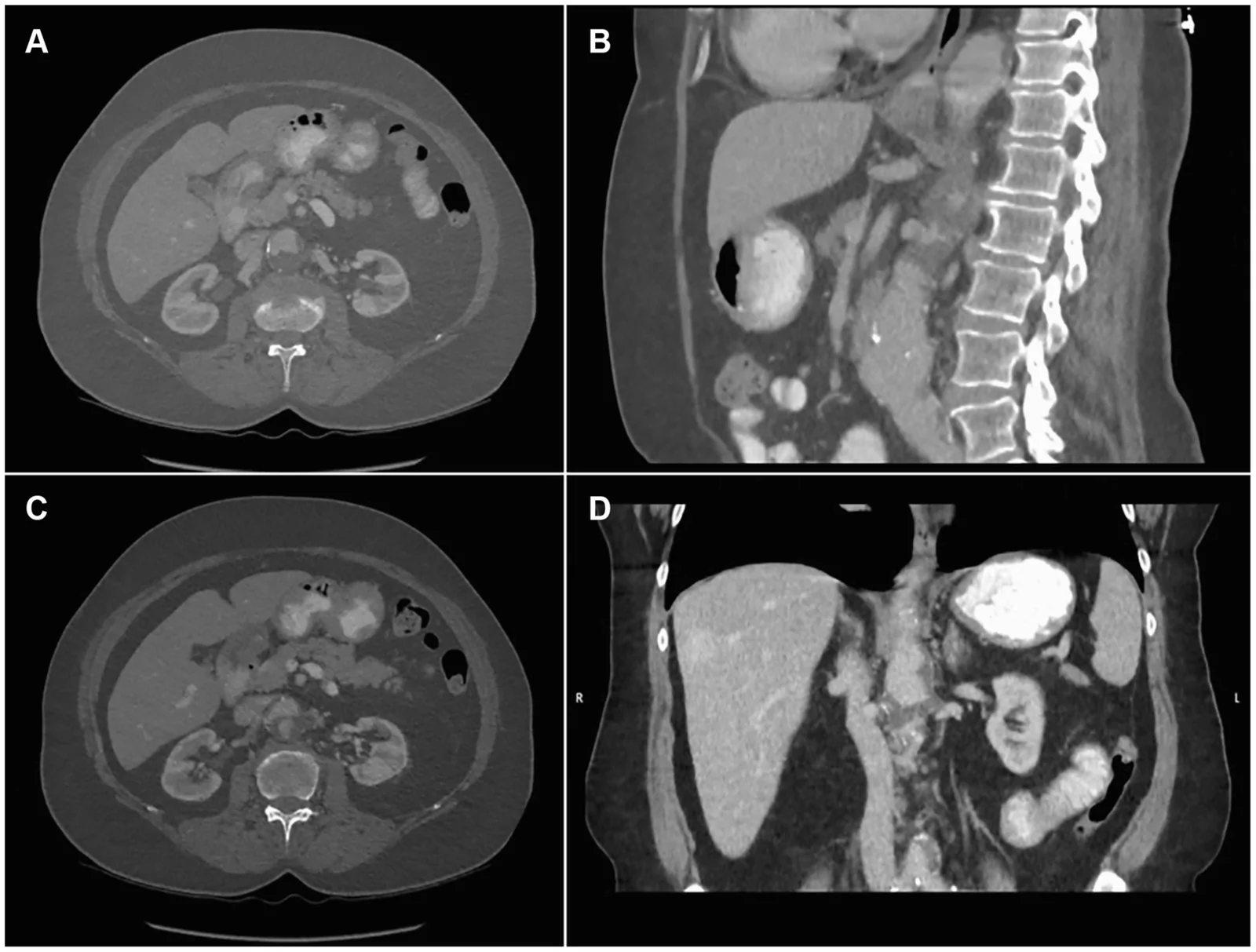
Preoperative computed tomography scan showing extensive perirenal thrombus in **(A** and **C)** proximal axial, **(B)** sagittal, and **(D)** coronal views.

**Fig 2:**
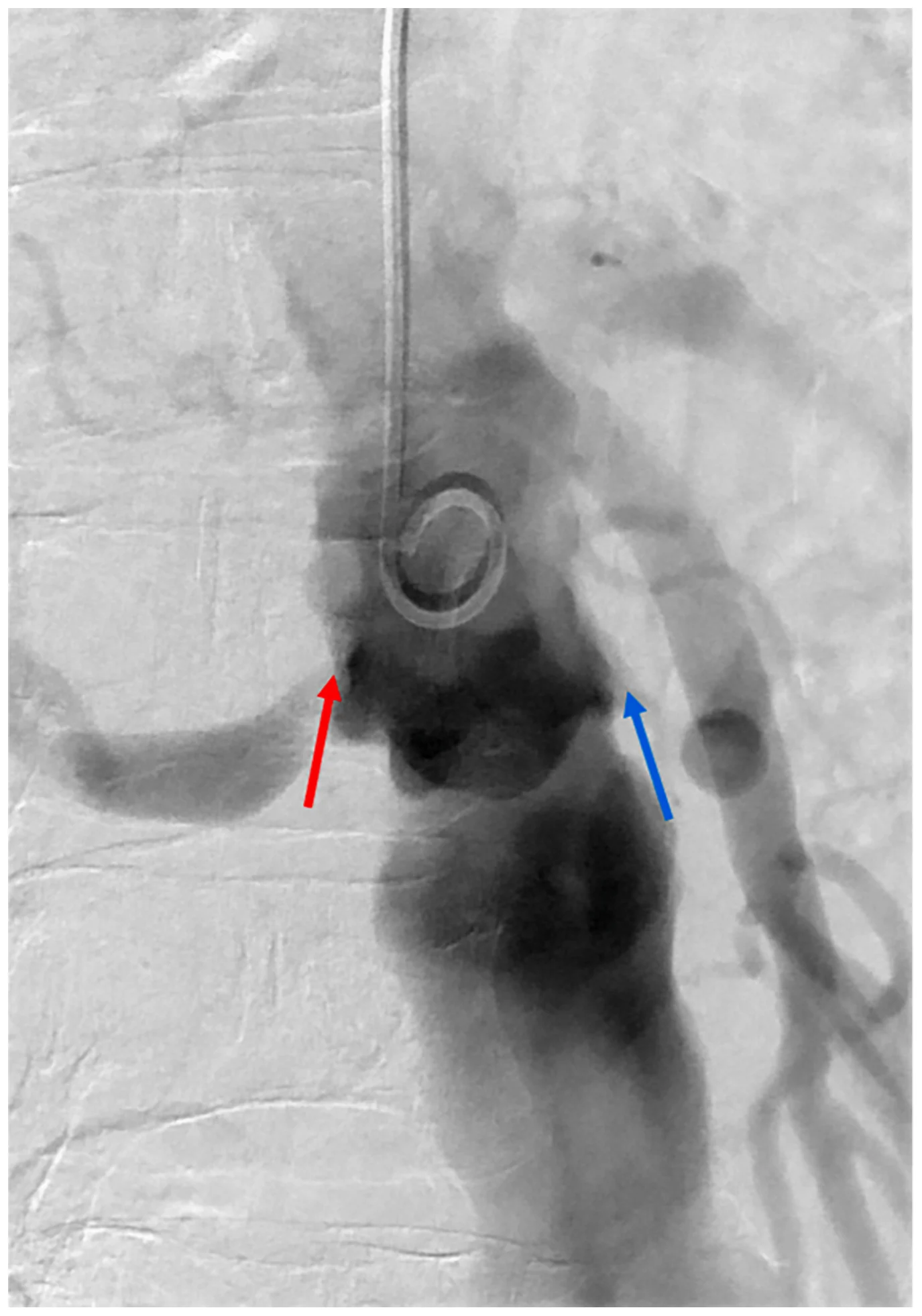
Preoperative angiogram showing extensive perivisceral aortic disease, with stenosis of the right renal artery (*red arrow*) and near occlusion of the left renal artery (*blue arrow*).

A thoracobirenal bypass was constructed using a Hemashield Gold bifurcated Dacron graft (Getinge). The proximal anastomosis was created on the descending thoracic aorta using a side-biting clamp, at approximately the T10-11 vertebral level. During this, the patient remained hemodynamically stable with no concerns for dissection. The graft was routed through the diaphragmatic hiatus, with the left limb anastomosed end-to-end to the left renal artery. Because of limited exposure, from the initial retroperitoneal approach, the right renal artery was accessed via a transperitoneal approach, and the right limb was tunneled retroperitoneally and anastomosed in a similar fashion (Fig 3). Native renal artery stumps were oversewn. Completion Doppler examination demonstrated adequate low-resistance flow to both kidneys.

**Fig 3:**
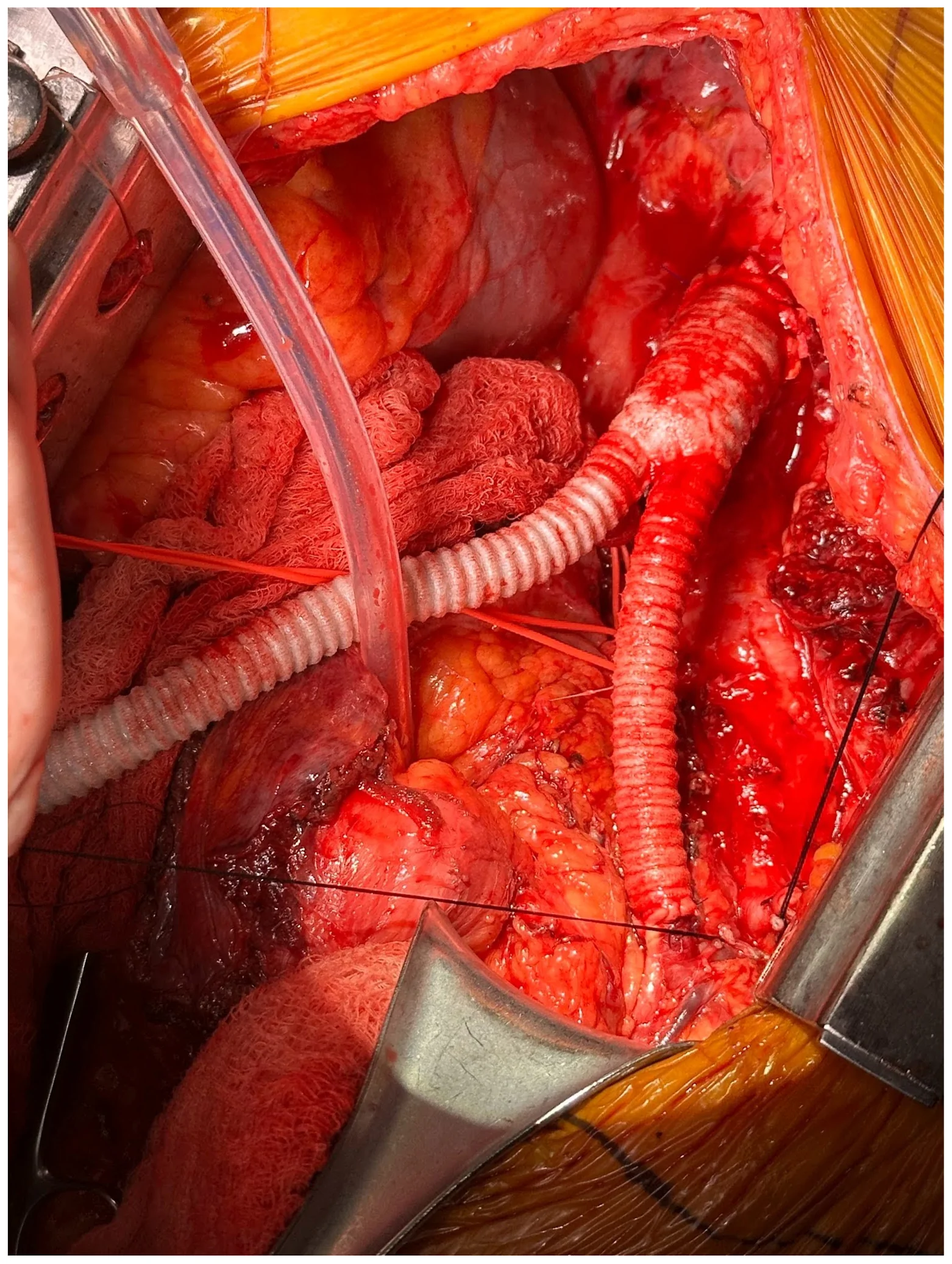
Bifurcated aortobirenal graft with left renal artery anastomosis (shown) and right renal artery limb (*off screen right*).

The patient remained intubated until postoperative day 5 because of severe atelectasis and was discharged home 1 week later. At 2-week follow-up, renal function had significantly improved, with normalization of serum creatinine and a glomerular filtration rate greater than 60 mL/min/1.73 m^2^. Follow-up computed tomography demonstrated a widely patent bypass graft (Fig 4) with preserved renal perfusion, with a descending thoracic aortic dissection extending into the visceral segment (Fig 5). This type B aortic dissection was likely related to the proximal thoracic aortic clamping.

**Fig 4:**
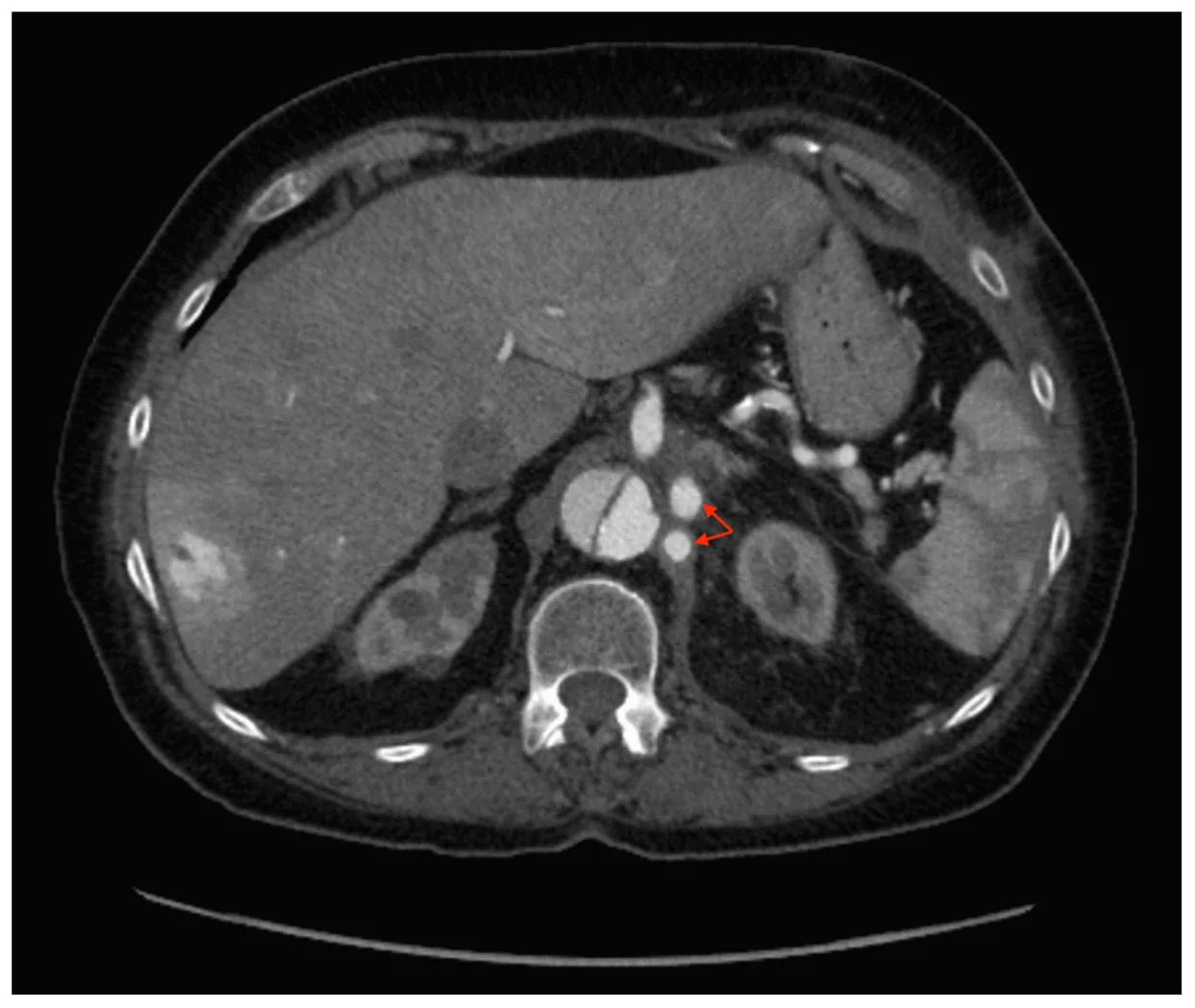
Postoperative computed tomography angiogram showing dissection flap extending to the perivisceral segment of the aorta and patent renal artery limbs (*red arrow*).

**Fig 5:**
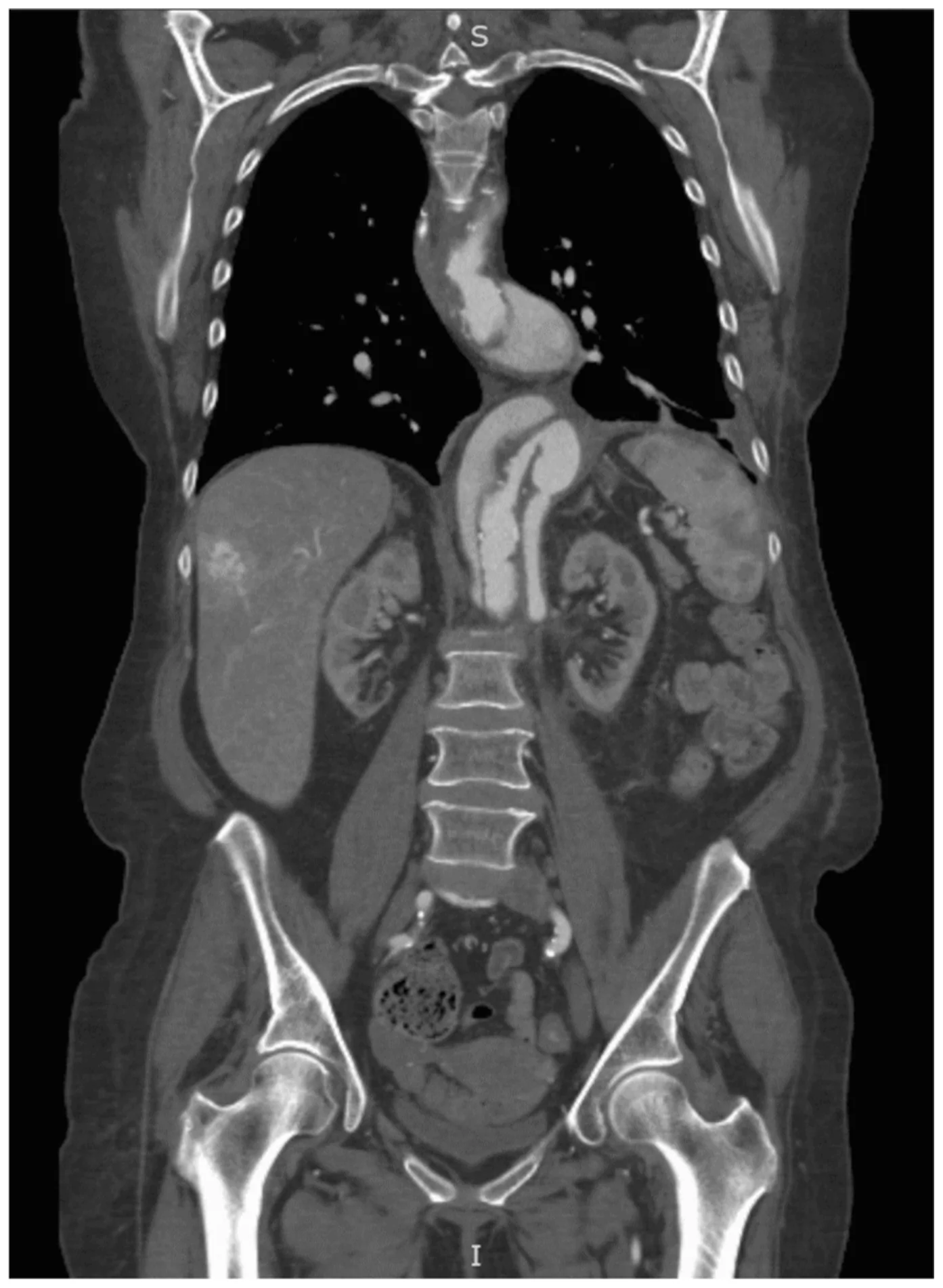
Postoperative computed tomography scan showing aortic dissection and the proximal anastomosis of the bilateral renal artery bypass.

The patient remained asymptomatic and hemodynamically stable with well-controlled blood pressure. At 2-year follow-up, the patient remained asymptomatic with preserved renal function, duplex ultrasound imaging confirmed continued patency of the thoracobirenal bypass graft, and there was no evidence of dissection propagation or aneurysmal degeneration.

## Discussion

Embolic disease resulting in organ compromise from aortic and perivisceral sources remains a challenging pathology, particularly given the lack of clear treatment guidelines.^3^ In this patient, conservative management with anticoagulation, smoking cessation, and best medical therapy failed to prevent progressive renal dysfunction. Given the extensive perivisceral thrombus burden, renal artery involvement, and ongoing decline in renal function, intervention was necessary to limit further solid organ injury. Although endovascular approaches are increasingly used for complex aortic pathology, their role in the setting of aortic thrombus remains controversial because of the risk of embolization.^3^^,^^4^ Jeyabalan et al^5^ describe the potential for visceral or peripheral embolization resulting from wire and catheter manipulation, angioplasty, or stent deployment. In this case, thrombus extension into the renal artery origins significantly increased the risk of renal embolization with endovascular techniques.^6^ Balloon embolectomy with temporary visceral occlusion has been described as a strategy to mitigate embolic risk; however, residual friable thrombus may persist, particularly in the setting of extensive chronic disease.^1^ Given the patient’s compromised renal function and significant thrombus burden, the risk of further embolization outweighed the risks associated with open repair.

The open approach provided several advantages. Direct exposure allowed accurate assessment of the extent of disease, which was more extensive than what was appreciated on preoperative imaging. Although aortic and visceral endarterectomy was performed, the renal artery ostia remained significantly compromised, making isolated endarterectomy insufficient. This intraoperative finding necessitated conversion to a thoracobirenal bypass to achieve durable renal revascularization. Although reports of renal bypass for perivisceral aortic thrombus are limited, similar strategies have been described in other complex aortic pathologies, including coral reef aorta and ascending aortic thrombus, where extensive disease involving the visceral segment may require bypass or reimplantation.^2^^,^^7^, ^8^, ^9^ Kim et al^9^ demonstrated successful management of extensive coral reef aortic disease using bypass from the distal thoracic aorta with visceral revascularization, highlighting the limitations of endarterectomy alone in such settings. In this context, conversion to a thoracobirenal bypass provided an effective and individualized solution for this patient’s extensive disease.

Postoperative surveillance demonstrated a nonflow limiting descending thoracic aortic dissection extending into the visceral segment. This was likely related to proximal clamping or intimal disruption. The patient remained asymptomatic, hemodynamically stable, and without evidence of malperfusion or aneurysmal degeneration. Accordingly, the dissection was managed conservatively with strict blood pressure control and imaging surveillance, consistent with established management of uncomplicated type B aortic dissection.^10^

If future intervention becomes necessary due to aneurysmal degeneration, progressive dissection, malperfusion, or persistent false lumen expansion, endovascular repair would be considered. Available strategies include thoracic endovascular aortic repair, septal fenestration, and adjunctive bare metal stenting. The provisional extension to induce the complete attachment (PETTICOAT) technique has been described as a method to improve distal aortic remodeling by combining proximal entry tear coverage with distal bare metal stent support. This approach may enhance true lumen expansion, reduce false lumen perfusion, and decrease the risk of late complications such as aneurysmal degeneration or reintervention.^11^^,^^12^ In the present case, however, the absence of symptoms or high-risk imaging features supports continued surveillance.

## Conclusions

This case describes severe perivisceral aortic thrombus with bilateral renal artery involvement and progressive ischemic nephropathy managed with open endarterectomy and extra-anatomic thoracobirenal bypass. It highlights the limitations of endovascular therapy in the setting of extensive aortic thrombus and emphasizes the importance of intraoperative flexibility when preoperative imaging underestimates disease burden. Thoracobirenal bypass may be a useful option in selected patients when endarterectomy alone is insufficient and endovascular intervention carries unacceptable embolic risk. Continued surveillance is essential, particularly when postoperative aortic complications such as dissection are identified. Larger studies are needed to define the durability, patency, and broader clinical utility of this approach.

## Funding

None.

## Disclosures

None.
